# Cherax (Astaconephrops) pulcher, a new species of freshwater crayfish (Crustacea, Decapoda, Parastacidae) from the Kepala Burung (Vogelkop) Peninsula, Irian Jaya (West Papua), Indonesia

**DOI:** 10.3897/zookeys.502.9800

**Published:** 2015-05-04

**Authors:** Christian Lukhaup

**Affiliations:** 1 Waldstrasse.5a, 66999 Hinterweidenthal, Germany

**Keywords:** Crustacea, Decapoda, Parastacidae, Cherax (Astaconephrops) pulcher, new species, freshwater crayfish, Hoa Creek, Teminabuan Region, Irian Jaya, Indonesia, West Papua

## Abstract

A new species, Cherax (Astaconephrops) pulcher **sp. n.**, from Hoa Creek, close to the village Teminabuan in the southern-central part of the Kepala Burung (Vogelkop) Peninsula, West Papua, Indonesia, is described, figured and compared with the morphologically closest species, *Cherax boesemani* Lukhaup & Pekny, 2008.

## Introduction

**Figure 1.**
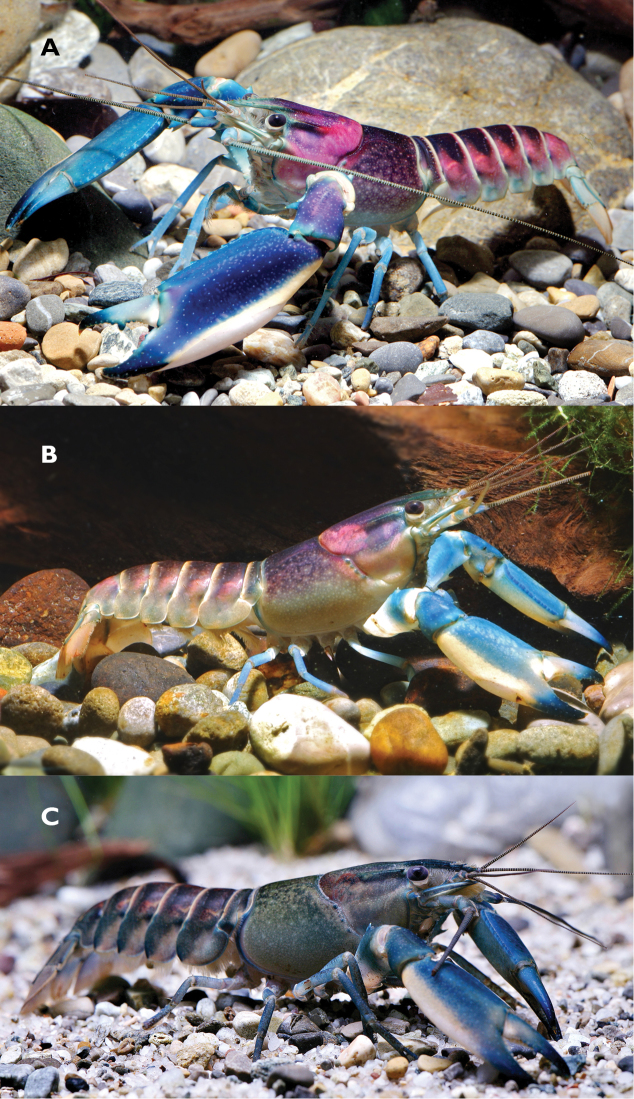
Cherax (Astaconephrops) pulcher sp. n. **A** adult male from Aquarium Dietzenbach **B** immature male from Hoa Creek, West Papua **C** female from aquarium import (not listed in material examined) from Indonesia.

The crayfishes of the island of New Guinea were extensively studied by Holthuis (1949, 1956, 1958, 1982, 1986, 1996), with recent additions by Lukhaup and Pekny (2006, 2008a) and Lukhaup and Herbert (2008). Nevertheless, over the last decade, there has been an increasing number of colourful crayfish sold in the ornamental fish trade in Europe, North America, and Asia under the names *Cherax* ”Hoa Creek”, ”Blue Moon”, and “Irian Jaya” presumed to represent a further undescribed species from New Guinea (Lukhaup and Pekny 2008b). The two most common and popular colour forms are: (1) a white, blue and violet morph with blue and white chelae (Fig. 1A–B); and (2) a greenish grey morph with blue and white chelae (Fig. 1C). While they are clearly species of *Cherax*, a large genus of freshwater crayfish occurring in Indonesia (West Papua), Papua New Guinea and Australia (Ahyong 2014), their exact provenances could not be ascertained, with dealers claiming originated from Sorong (West Papua) and other places in the area that could not be confirmed. In the present contribution, this species is described as new to science and shown to be native to the Teminabuan region of the Kepala Burung (Vogelkop) Peninsula West Papua, Indonesia. The new species, Cherax (Astaconephrops) pulcher sp. n. differs from all other crayfish of this subgenus in the shape of its chelae, shape of body and also in its coloration.

Abbreviations used: RMNH = Rĳksmuseum van Natuurlĳke Historie (= Naturalis Biodiveristy Center, Leiden). TL = Total length, CL = Carapace length.

## Systematics

### Family Parastacidae Huxley, 1879 Genus *Cherax* Erichson, 1846

#### Cherax (Astaconephrops) pulcher sp. n.

Taxon classificationAnimaliaDecapodaParastacidae

http://zoobank.org/C7C4B1F7-E5C3-45B2-ADC3-DE05EF489EE4

**Figure 2.**
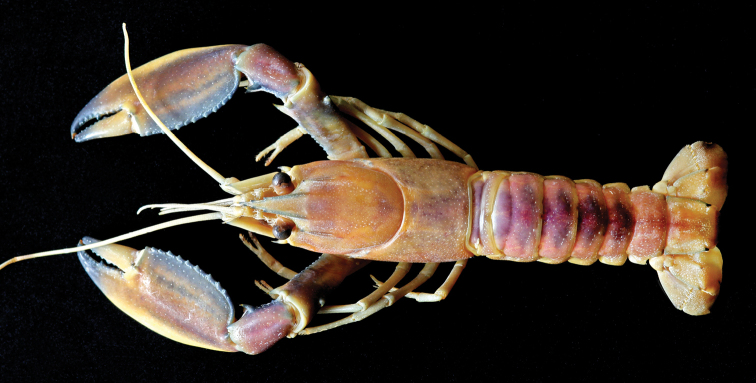
Cherax (Astaconephrops) pulcher sp. n. holotype male (RMNH.CRUS.D.57217).

**Figure 3.**
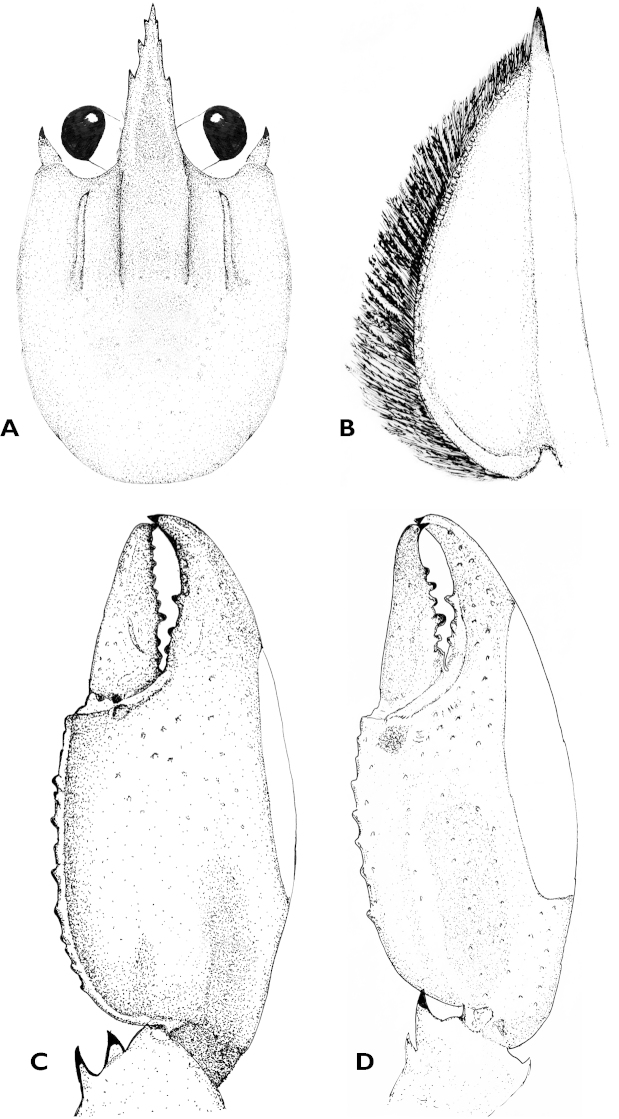
Cherax (Astaconephrops) pulcher sp. n. holotype male (RMNH.CRUS.D.57217) **A** dorsal view carapace **B** scaphocerite **C** dorsal view right chelae **D** ventral view left chelae.

**Figure 4.**
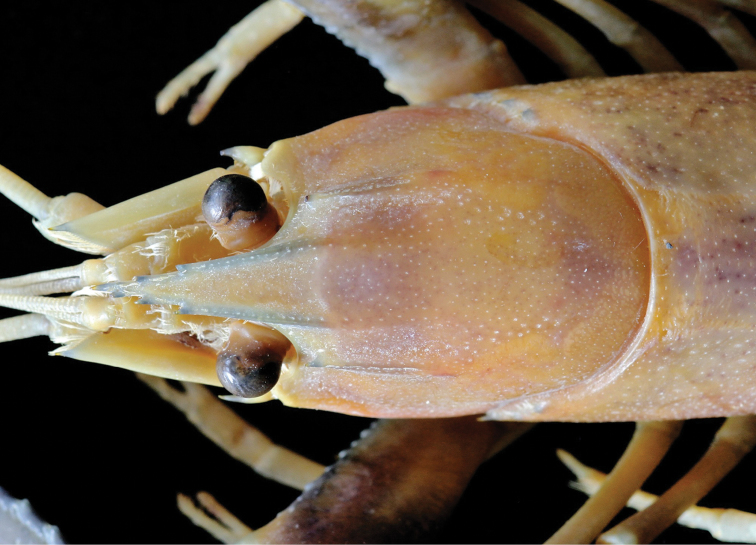
Cherax (Astaconephrops) pulcher sp. n. holotype male (RMNH.CRUS.D.57217) dorsal view of rostrum.

**Figure 5.**
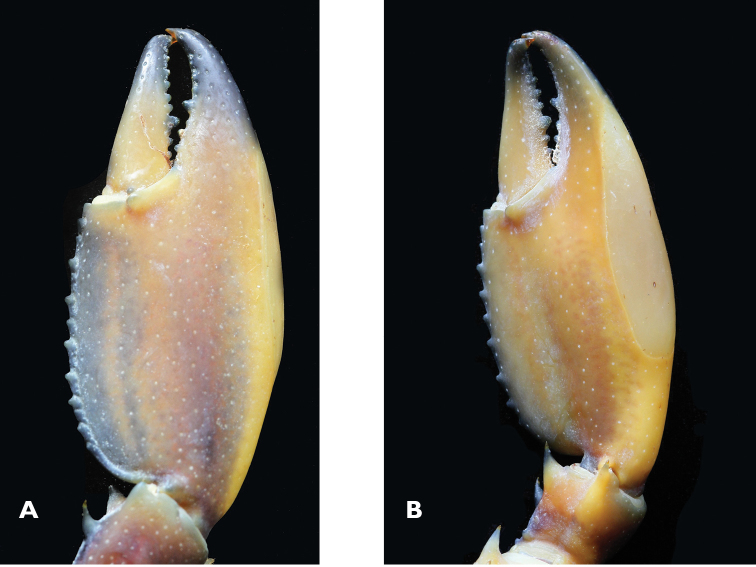
Cherax (Astaconephrops) pulcher sp. n. holotype male (RMNH.CRUS.D.57217) **A** right first chela, dorsal aspect **B** left first chela, ventral aspect.

Figs 1 , 2 , 3 , 4 , 5

##### Type material.

Holotype: male (TL) 96 mm) (RMNH.CRUS.D.57217), Hoa Creek, Teminabuan region, Kepala Burung (Vogelkop) Peninsula, West Papua, Indonesia, collector unknown, 5 October 2002. Paratypes: 1 male (TL 94 mm), 1 female (TL 90 mm) (RMNH.CRUS.D.57218), same data as holotype. All animals collected by and exported through Maju Aquarium, Jakarta, Indonesia.

##### Non-type material.

9 males (TL 83–98 mm), 1 female (TL 83 mm), from Aquarium Dietzenbach in Germany, 5 April 2004.

##### Description of male holotype

(Figs 2–5). Body and eyes pigmented. Eyes not reduced.

Body subovate, slightly compressed laterally. Pleon narrower then cephalothorax (width 17 mm and 20 mm respectively). Rostrum (Fig. 3A) slender, reaching about to end of ultimate antennular peduncle and about three times as long as wide (width 5 mm at base, length 14 mm). Upper surface smooth, few scattered setae present at the tip of the rostrum; lateral margins of rostrum almost straight in basal part, distally rather strongly tapering towards apex. Margins strongly elevated continuing in rostral carinae on carapax. Lateral rostral margin bearing 3 prominent teeth in distal half, on right side the third distal tooth (from base) is smaller than the others, few short hairs are present on outer margins. Rostral carinae extending as slight elevation posteriorly on carapace fading just before reaching cervical groove. Postorbital ridges well developed terminating in slightly upturned corneous spines anteriorly, fading at two-thirds of occipital carapace length, posteriorly. Scaphocerite (Fig. 3B) broadest at midlength, convex in distal part becoming narrower in basal part; thickened lateral margin terminating in large corneous spine. Right scaphocerite 11 mm long and 4 mm wide. Antennulae and antennae typical for the genus. Mouthparts typical for the genus.

Eyes rather large; cornea globular, darkly pigmented, about as long as eyestalk; eyestalk slightly narrower than cornea. Epistome broadly triangular, anteriorly becoming lance-shaped, scattered setae present, with corneous spine at anterior tip, lateral surface with small tubercles; central surface smooth, excavate.

Coxocerite of antennal peduncle with acute tooth anteriorly; basicerite with strong lateral spine. Cervical groove distinct, non-setose. Surface anterior to cervical groove smooth, anterior branchial margin at junction with cervical groove with 3, anteriorly directed, rather closely set spines just posterior to groove, uppermost at level of antennae. Areola length 13 mm narrowest width 9 mm. Length of areola 34.6% of total length of carapace (45 mm).

First pereopods equal in form and size. Right chelae (Fig. 3) 41 mm long and 8 mm high, 17 mm wide, strongly compressed. Fingers shorter than palm (dactylus 16 mm long). Dactylus broad at base, tapering slightly towards tip, becoming about 1/3 as broad as at base. Tip with sharp, corneous, hooked tooth pointing outwards at an angle of 45°. Cutting edge of dactyl with a continuous row of rather small granular teeth and one large prominent tooth at about middle of cutting edge. Ventral and dorsal surface of movable finger with scattered punctuation. Fixed finger triangular, merging gradually into palm, ending in sharp, corneous, hooked tooth, standing almost perpendicular to axis of finger. Upper surface of palm practically smooth, slightly pitted, more densely pitted at margins. Chelae non-setose except for ventral cutting edge of fixed fingers. Setae short, present only in posterior part. First cheliped of adult male with soft, decalcified swollen area (Fig. 5A) in distal part of the lower margin,characteristic of the subgenus. Soft area extending from the first third of the fixed finger to the distal third of the fixed finger covering slightly more than half (22 mm) of the distal part of the lower margin. Carpus with slightly elevated part ventral, ending in a corneous spine. Three prominent spines present at the proximal part of the carpus. Ventral surface smooth and pitted but with median portion elevated into a prominent, broad ridge ending in corneous spine. Dorsal surface of merus smooth, with slight excavation in middle part, with a distomesial spine and a tubercle on the dorsolateral surface and dorsodistal margin. Dorsolateral margin with 1 corneous spine. Ventral surface with 3 large corneous spines. Ischium smooth with single spine on ventral surface.

Second pereopod reaching anteriorly about to middle of scaphocerite and a bit further when stretched. Fingers as long as palm, of same height. Short setae present on dactyl and fixed finger. Carpus slightly longer than palm. Merus about 1.5 times longer than carpus. Ischium about half as long as merus.

Third pereopod overreaching second. Fingers shorter than palm.

Fourth pereopod reaching distal margin of scaphocerite. Dactylus with corneous tip. Short scattered setae present. Propodus more than twice as long as dactylus, about 1.5 times as long as carpus; somewhat flattened, carrying many stiff setae on lower margin. Merus just slightly longer than propodus.

Fifth pereopod similar to fourth, slightly shorter.

Dorsal surface of pleon smooth in median region; pleura smooth, slightly pitted becoming densely pitted on sixth somite and telson. Telson with posterolateral spines. Uropodalm protopod with distal spine on mesial lobe. Exopod of uropod with two well defined spines. one distal spine on mesial lobe, with prominent median rib ending in a spine in middle of uropod. Posterior margin of proximal segment of exopod of uropod with row of small spines overlapping diaresis.

##### Description of paratype female

**Figure 6.**
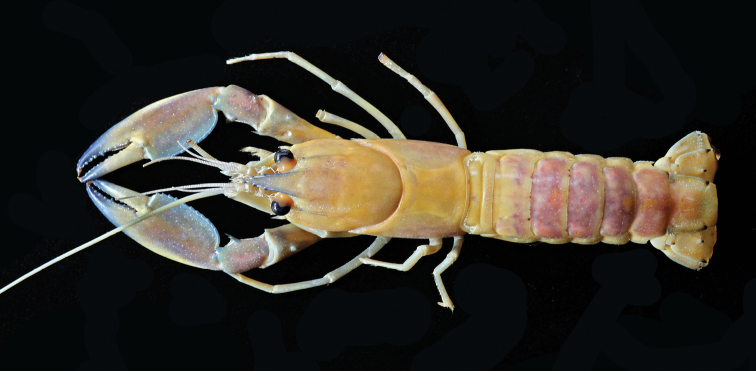
Cherax (Astaconephrops) pulcher sp. n., paratype female (RMNH.CRUS.D.57218).

(Fig. 6). Chela of first pereiopods equal, about 3 times as long as broad (30 mm and 11 mm respectively), with no decalcified areas on lower margin. Mesial margin of palm slightly elevated, forming slender serrated ridge with row of 9 small granular teeth. Cutting edge of dactyl with rather small granular teeth in posterior part and one slightly larger tooth in about middle. Cutting edge of fixed finger with small granules and one slightly larger granules. Small scattered short setae visible along ventral cutting edge of chelae, more dense in posterior area. Cervical groove distinct, non setose. 3 spines present on lateral surface of cephalothorax. At level of antenna, two weakly developed anteriorly directed spines present. Pleon just slightly narrower than cephalothorax (widths 16 mm and 17 mm respectively).

##### Remarks.

In young males, the first chelae resembles those of the female or is slightly broader. A young male of TL 74 mm (non type material )has the decalcified area small, in the process of developing, present on both chelae, about 10 mm long.

##### Size.

The males examined have a carapace length of 35–47 mm, and a total length of 75–98 mm (n = 11); the females have a carapace length of 37–39 mm and a total length of 83–90 mm (n = 2).

##### Coloration.

The living animals (Fig. 1) are coloured as follows: Chelae light blue to dark blue, decalcified swollen area in distal part of the lower margin white or cream coloration, rostrum greenish blue. Anterior part of the cephalothorax pinkish to striking pink fading laterally to a greenish grey. Walking legs light blue to dark blue. Dorsal pleon dark blue to black becoming pinkish grey and cream coloured to the margins. Some individuals greenish-grey with few pinkish spots on pleon. Chela light blue with cream coloured margins. Walking legs bluish. Distal margin of tail-fan cream to pinkish.

##### Systematic position.

The presence of a decalcified area on the lower margin of the chelae of the first pereiopods in adult males shows that the new species belongs to the subgenus *Astaconephrops*. Seven species/subspecies are known from New Guinea: Cherax (Astaconephrops) lorentzi lorentzi Roux, 1911; Cherax (Astaconephrops) lorentzi aruanus Roux, 1911; Cherax (Astaconephrops) minor Holthuis, 1996; Cherax (Astaconephrops) monticola Holthuis, 1950; Cherax (Astaconephrops) misolicus Holthuis, 1949; Cherax (Astaconephrops) albertisii (Nobili, 1899) and Cherax (Astaconephrops) boesemani Lukhaup & Pekny, 2008.

##### Etymology.

The name is derived from the Latin “pulcher” meaning beautiful, alluding to the colourful appearance of the species.

##### Ecology.

Known only from the Hoa Creek and the general Hoa Creek Drainage area. The water is clear, and has pH 6.6. Currents are strong in the narrower parts of the creek, including the upper reaches. The substrate of the creek is rocky, and mostly covered with sand, stones and large rocks. To improve the knowledge of the distribution of the species more collecting trips are necessary.

## Discussion

With the current description of the new species of *Cherax* from the Teminabuan Region, Kepala Burung, West Papua, Indonesia, 19 species of *Cherax* are now known from Indonesia (Lukhaup and Pekny 2008b). Other than the very distinctive and different colour pattern, the new species is morphologically very similar to *Cherax boesemani* Lukhaup & Pekny (2008a). *Cherax boesemani* differs from *Cherax pulcher* sp. n. in size, coloration, the shape of the chelae and the shape of the cephalothorax. *Cherax boesemani* reaches a TL up to 25 cm while *Cherax pulcher* sp. n. reaches a maximum length up to 12 cm body length. (Lukhaup and Pekny 2014). The body colour of *Cherax boesemani* is usually a dark red to brown, some individuals orange red, greenish gray. The chelae of *Cherax boesemani* are variable in colour but mostly red to black with pinkish margins and black tips, sometimes beige, olivaceous beige, olivaceous brown, olivaceous green, reddish brown, purplish. The chelae coloration of *Cherax pulcher* sp. n. is light blue to dark blue and cream or white distally. The chelae of *Cherax boesemani* are 5.4 times as long as high, while in contrast in *Cherax pulcher* sp. n. they are 5.1 times as long as high.

*Cherax boesemani* is a species having a narrower areola, thus better adapted to bodies of standing, warmer water, like pools or lakes. In contrast, *Cherax pulcher* sp. n. has a wider areola and the body shape compared to *Cherax boesemani* is more slender, thus it is adapted to fast flowing water with higher dissolved oxygen levels. In general, lake crayfish also get bigger in size then the creek or river species.

The type localities of *Cherax boesemani* (see Lukhaup and Pekny 2008a) and *Cherax pulcher* sp. n. are relatively close, although they are in separate valleys. *Cherax boesemani* was found about 22 km east of the type locality of *Cherax pulcher* sp. n. The habitat of *Cherax pulcher* sp. n. is clear, fast flowing creeks while *Cherax boesemani* is found only in Ajamaru Lake and the Ajamaru River.

It is also necessary to briefly comment on the possible threats faced by the new species. As *Cherax pulcher* sp. n. is collected in large numbers for the global aquarium trade, as well as for food for the growing local population, the crayfish population will invariably be adversely impacted. According to local collectors in the area and the city of Sorong, the populations of the species have been decreasing in the last few years. Clearly, the continued collection of these crayfish for the trade is not a sustainable practice, and if the popularity of the species continues, a conservation management plan will have to be developed, including a captive breeding program.

## Supplementary Material

XML Treatment for Cherax (Astaconephrops) pulcher
